# Highly Sensitive and Specific Detection of Rare Variants in Mixed Viral Populations from Massively Parallel Sequence Data

**DOI:** 10.1371/journal.pcbi.1002417

**Published:** 2012-03-15

**Authors:** Alexander R. Macalalad, Michael C. Zody, Patrick Charlebois, Niall J. Lennon, Ruchi M. Newman, Christine M. Malboeuf, Elizabeth M. Ryan, Christian L. Boutwell, Karen A. Power, Doug E. Brackney, Kendra N. Pesko, Joshua Z. Levin, Gregory D. Ebel, Todd M. Allen, Bruce W. Birren, Matthew R. Henn

**Affiliations:** 1 Broad Institute of MIT & Harvard, Cambridge, Massachusetts, United States of America; 2 Department of Biostatistics, Harvard University, Boston, Massachusetts, United States of America; 3 Ragon Institute of MGH, MIT and Harvard, Boston, Massachusetts, United States of America; 4 Department of Pathology, University of New Mexico School of Medicine, Albuquerque, New Mexico, United States of America; University of California San Diego, United States of America

## Abstract

Viruses diversify over time within hosts, often undercutting the effectiveness of host defenses and therapeutic interventions. To design successful vaccines and therapeutics, it is critical to better understand viral diversification, including comprehensively characterizing the genetic variants in viral intra-host populations and modeling changes from transmission through the course of infection. Massively parallel sequencing technologies can overcome the cost constraints of older sequencing methods and obtain the high sequence coverage needed to detect rare genetic variants (<1%) within an infected host, and to assay variants without prior knowledge. Critical to interpreting deep sequence data sets is the ability to distinguish biological variants from process errors with high sensitivity and specificity. To address this challenge, we describe *V-Phaser*, an algorithm able to recognize rare biological variants in mixed populations. *V-Phaser* uses covariation (i.e. phasing) between observed variants to increase sensitivity and an expectation maximization algorithm that iteratively recalibrates base quality scores to increase specificity. Overall, *V-Phaser* achieved >97% sensitivity and >97% specificity on control read sets. On data derived from a patient after four years of HIV-1 infection, *V-Phaser* detected 2,015 variants across the ∼10 kb genome, including 603 rare variants (<1% frequency) detected only using phase information. *V-Phaser* identified variants at frequencies down to 0.2%, comparable to the detection threshold of allele-specific PCR, a method that requires prior knowledge of the variants. The high sensitivity and specificity of *V-Phaser* enables identifying and tracking changes in low frequency variants in mixed populations such as RNA viruses.

## Author Summary

New sequencing technologies provide unprecedented resolution to study pathogen populations, such as the single stranded RNA viruses HIV, dengue (DENV), and West Nile (WNV), and how they evolve within infected individuals in response to immune, therapeutic, and vaccine pressures. While these new technologies provide high volumes of data, these data contain process errors. To detect biological variants, especially those occurring at low frequencies in the population, these technologies require a method to differentiate biological variants from process errors with high sensitivity and specificity. To address this challenge, we introduce the *V-Phaser* algorithm, which distinguished the covariation of biological variants from that of process errors. We validate the method by measuring how frequently it correctly identifies variants and errors on actual read sets with known variation. Further, using data derived from a patient following four years of HIV-1 infection, we show that *V-Phaser* can detect biological variants at frequencies comparable to approaches that require prior knowledge. *V-Phaser* is available for download at: http://www.broadinstitute.org/scientific-community/software.

## Introduction

Genetic differences can arise among individual viral particles within an infected host, and detecting these viral genetic variants can reveal how viruses adapt to challenges such as host immune responses, antiviral medications, and transmission bottlenecks. However, detecting rare variants is difficult with existing sequencing technologies due to low sensitivity, high error rates, and/or poor scalability. For example, bulk-sequencing approaches generate a consensus assembly, but they have limited sensitivity to detect intra-host variation [1]. One approach to increase sensitivity is to amplify and clone selected fragments of viral nucleic acids into proliferating targets that are subsequently isolated and sequenced [2], but this method has a higher false positive rate and poor scalability. To reduce errors, the single genome amplification (SGA) method isolates individual viral genomes through dilution, and then amplifies and sequences each genome individually to minimize introduced errors [3]–[5], although scalability remains an issue. Rare variant detection requires deep coverage that is not cost-effective with current methods of cloning or SGA. To address scalability, massively parallel sequencing technologies can isolate and sequence individual DNA or cDNA molecules en masse from the population of viral genomes and generate millions of short read sequences that can increase the sensitivity and decrease the cost to detect variants [6], [7]. Still, increased error rates can somewhat impact potential gains in sensitivity. Here, we report on a novel method to detect rare variants that increases sensitivity even in the presence of process errors.

Detecting biological variants involves not only finding them, which deep sequencing technologies can do with high sensitivity, but also differentiating them from process (i.e. amplification or sequencing) errors. One way to do this is to compare variants to a distribution of errors. For example, several authors have reported using a Poisson or binomial probability model to define the error distribution, and they can call candidates that fall outside the distribution variants [8]–[13]. These models, however, assume that all bases have equal quality scores, where the base quality score is a measure of how accurate the base call is. This assumption is invalid for bases measured by massively parallel sequencing technologies, as Brockman et al. [14] have shown, since base quality can vary by several criteria; in fact, sequencing technologies take criteria such as these into account when assigning base quality scores. To avoid this assumption, probability models can incorporate base quality scores. Such probability models exist in tools that call single nucleotide polymorphisms (SNPs) in human and other diploid genomes, including MAQ [15], SoapSNP [16], Unified Genotyper [17], SNVMix [18], or Slider [19]. In contrast, instead of an explicit error probability model, Hoffman et al. [20] compare variants to an empirical control data set. Archer et al. [21] and Rozera et al. [22] report methods that correct read sequences for suspected process errors prior to calculating variant frequencies. Archer et al. [21] use a k-mer mapping approach to position reads on a consensus template and refine alignments locally, and Rozera et al. [22] turn to heuristic rules to filter out errors based on cutoffs for base quality scores and other criteria. Both strategies avoid using an explicit probability model of error and hence assume that all process errors take a specific form, and that no biological variants take the same form as the process errors.

The above models separate variants from error using specific forms or heuristics or a probabilistic distribution. An alternative approach is to consider patterns of candidate variants. For example, Eriksson et al. [9] use Fisher's exact test to find patterns that occur more frequently than expected by chance to call variants. Refining this approach further, several authors probabilistically cluster patterns to infer variant haplotypes [9], [11], [12], [23]; the cluster centers are haplotypes, and process errors can be removed by collapsing variation within the cluster. Since patterns of variants are essentially groups of variants that occur at the same loci on multiple reads, i.e. in phase, we can analyze them together as a group of phased variants, and we can compare them to phased errors in the same pattern. Phased errors presumably occur much less frequently than errors in general, making it easier to recognize phased variants.

To address the challenge of calling rare genetic variants in diverse populations in the presence of error, we introduce *V-Phaser*, a single nucleotide variant calling tool that uses phase and quality filtering with a probability model that incorporates and recalibrates individual base quality scores. To increase sensitivity, *V-Phaser* looks not only for variants that fall outside the distribution of errors but also for patterns of variants in phase. To increase specificity, it incorporates individual base quality scores into a composite Bernoulli model that allows error rates to vary from base to base. It also uses a pre-processing filter to screen out low quality bases and improve the fit of the model. We calculate the theoretical gain in sensitivity of detecting variants using phase to increase specificity. We then validate *V-Phaser* on read sets with known variation generated by the 454 sequencing platform to estimate sensitivity and specificity. To determine the effect of each algorithmic step on performance, we evaluate the method with each of three features (phasing, recalibration, and filtering) turned off and compare these results to those achieved on the same data with several other viral variant callers. Finally, we use *V-Phaser* on data from a chronically HIV-1 infected subject to demonstrate its utility to detect low frequency variants in viral populations.

## Results

### Using phased variants to increase sensitivity

Variant calling algorithms typically use a probabilistic or empirical error model to define the distribution of errors, and they recognize those candidates that fall outside of this distribution as variants. We define the boundary between variants and errors to be the *variant detection threshold frequency* (*VDTF*). To this definition, we add the concept of phasing, where phased variants co-occur on the same reads, to distinguish *unphased VDTFs* from *phased VDTFs*, which separate phased variants from phased errors. *V-Phaser* uses both *phased* and *unphased VDTFs* to increase sensitivity. If errors are distributed uniformly at a rate *p*, we cannot use *unphased VDTFs* to find variants that occur below this rate no matter how deeply we sequence the population, since the *unphased VDTF* cannot fall below *p*. In contrast, paired errors occur at the much lower rate of *p* ^2^, and correlated variant pairs can be detected at much lower frequencies than *p*, so long as that frequency remains above *p* ^2^ and the depth of sequencing is sufficient. Theoretically, we can define *phased VDTFs* for any pattern of variants, but in practice the only patterns *V-Phaser* considers are paired variants in phase.

**Figure 1:**
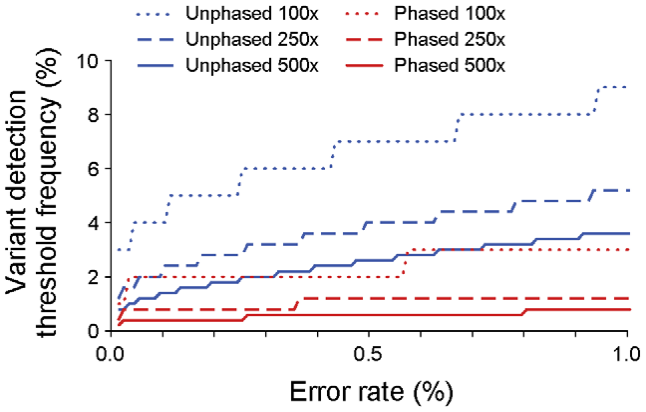
Phase increased sensitivity to detect variants. Phase increased sensitivity to detect variants, as seen over a range of error rates at coverages of 100-fold, 250-fold, and 500-fold. The *phased variant detection threshold frequency (VDTF)* is the lowest frequency of reads with variants at two specific loci that *V-Phaser* can distinguish from error among reads that span both loci. The *unphased VDTF* is the lowest frequency of one variant that *V-Phaser* can distinguish from error among reads that cover that locus. 100-fold *phased* sequence coverage achieves comparable detection thresholds as 500-fold *unphased*. We use Equation 7 to calculate the *phased* and *unphased VDTFs*. (See the Materials and Methods section for Equation 7 and its derivation.)

*V-Phaser* can call paired variants at a lower frequency using *phased VDTFs* compared to *unphased VDTFs*, and given comparable frequencies, *V-Phaser* can call phased variants at lower coverage. We can calculate *phased* and *unphased VDTFs* as a function of coverage and error rate (Figure 1). At any level of coverage and error rate, the *phased VDTF* is lower than the *unphased VDTF*. In addition, the *phased VDTF* remains relatively flat over a range of error rates, whereas the *unphased VDTF* manifests more dynamic range, which suggests that compared to the *unphased VDTF*, even if the probability model grossly misspecifies error rates, the *phased VDTF* is relatively robust.

**Figure 2:**
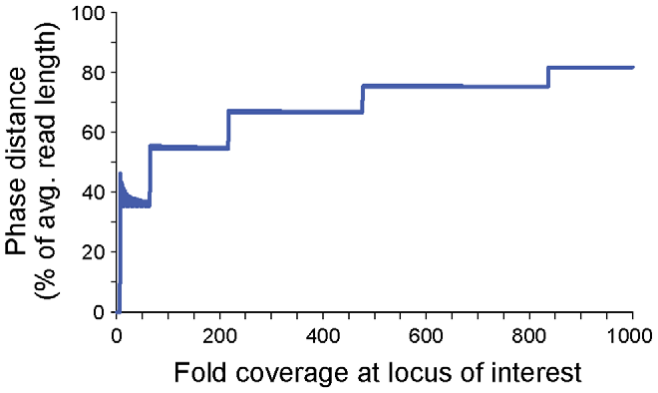
Phase distance approached length of average read as coverage increased. The *phase distance* was longer than half the average read length for loci covered more than 65-fold, and as coverage increased, it approached the length of the average read. The *phase distance* is a measure of how far apart phased variants can be and still be detected at lower frequencies than variants not in phase. We show the phase distance as a percentage of average read length.

*V-Phaser* detects variants in phase when they occur on the same reads, so to be on the same reads, variants need to be close to each other. When variants are close together, many of the reads that cover one variant will also cover the other variant, but when variants are farther apart, fewer and fewer reads will begin and end in just the right places to span both. At some point, the gain from phase will be offset by the loss of shared coverage. To capture this concept, we define the *phase distance* to be the farthest distance from a locus such that compared to the *unphased VDTF* at that locus, the *phased VDTF* is lower (i.e. more informative). If variants are farther apart than the *phase distance*, they do not have enough shared coverage to increase sensitivity. We show that the *phase distance* is longer than half of the average read length for coverage more than 65-fold, and as coverage increases, it approaches the length of the average read (Figure 2). Just as increasing coverage increases sensitivity to detect variants, it also increases the chances to detect phased variants that are farther apart.

### Pre-process filtering and recalibration of base quality scores to increase specificity

**Figure 3:**
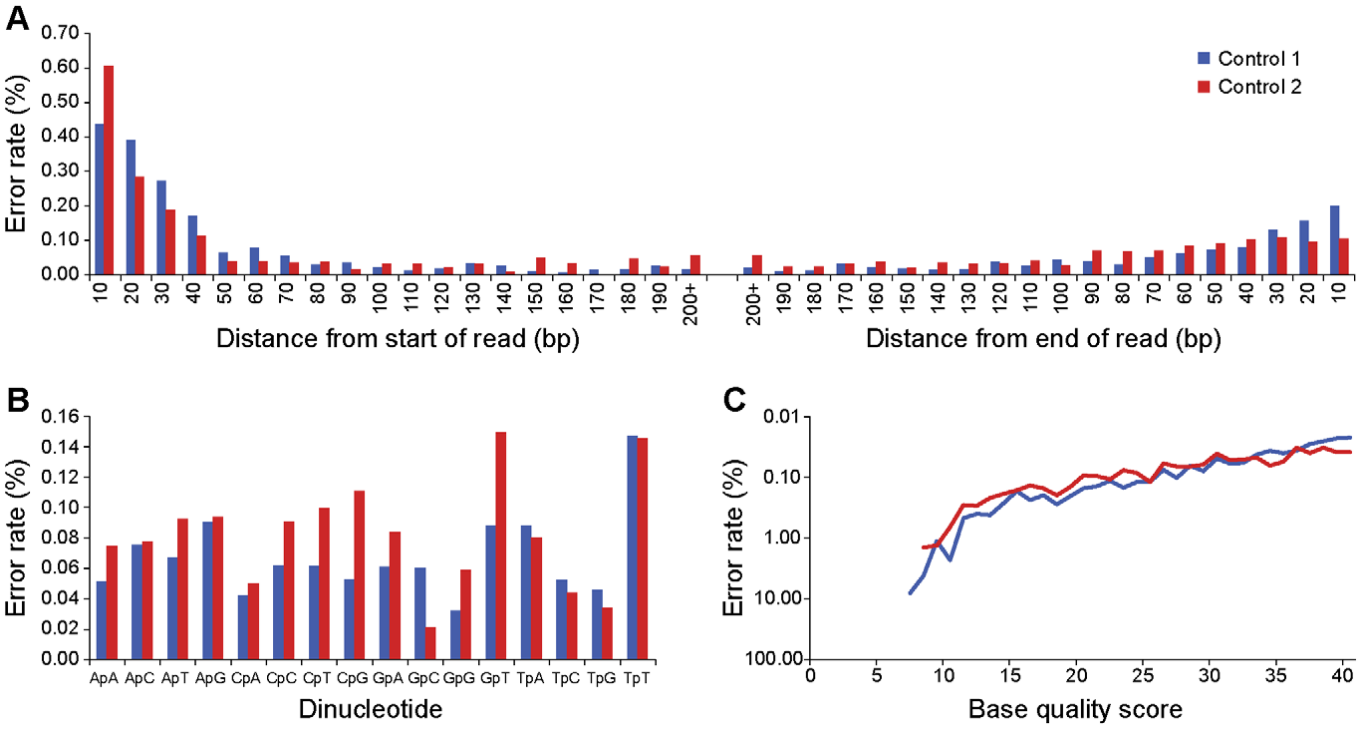
Error rates were not uniformly distributed. Error rates varied by (**A**) read position, (**B**) base transition, and (**C**) base quality score. We counted as errors any mismatches to the consensus assembly for each of the two runs in the control read set under the assumption that the NL-43 infectious clone had no diversity. We defined the read position relative to the beginning or end of the read, whichever was closer. We defined a base transition as a dinucleotide representing the transition from the preceding base to the current base, and we scored a transition as an error if the current base was a mismatch. Base quality scores came from the sequencing process.

Errors introduced by massively parallel sequencing technologies can be correlated, and models to detect correlated variants can also detect correlated errors, as well. On control read sets without variants, we found that errors vary with base quality score, the position of the base on the read, and the transition from the previous base (Figure 3 a–c). DePristo et al. [17] use recalibration equations in their Unified Genotyper to adjust for these associations and call SNPs. *V-Phaser* minimizes false positive correlated errors by filtering out errors and modeling the correlations among errors. First, as detailed in the Materials and Methods section, *V-Phaser* uses a read cleanup algorithm, *ReadClean454* [24], to identify and correct process errors in the reads and then utilizes a Neighborhood Quality Standard (NQS) criteria to filter out low quality bases. From the remaining high quality bases, *V-Phaser* builds an error probability model to adjust for correlations.

Modeling base quality well is the key to achieving high specificity, but highly variable viral sequences pose a difficult challenge. To estimate the parameters, models are often fit to highly conserved genomic regions without variation, but such regions do not exist for small, diverse viral genomes. Models can also be fit to empiric negative controls, but error rates can vary from lane to lane or from run to run. Instead, *V-Phaser* uses an expectation-maximization (EM) algorithm to iteratively fit its probability model as it calls variants. Initially, *V-Phaser* treats all mismatches as errors and estimates the parameters accordingly using the recalibration equations of the Unified Genotyper [17]. In the E step, *V-Phaser* uses the model to calculate the *VDTFs* to call variants and remove them from the error list. Then in the M step, *V-Phaser* updates the parameters to the model. *V-Phaser* iterates until the number of variants called stabilizes.

### Validating *V-Phaser*

To evaluate *V-Phaser*'s performance, we used read sets with known variability generated by the 454 FLX sequencing platform. Using these control data, we validated the variants called by the comprehensive algorithm and also evaluated the contribution of each core component of *V-Phaser* to the model's sensitivity and specificity. First, we assessed the effect of using or not using phased variants by invoking a version of *V-Phaser* that only used *unphased VDTFs* to identify variants. Second, we measured the effect of using individual base quality scores utilizing a version of *V-Phaser* that estimates two uniform error rates, for homopolymer and nonhomopolymer regions. Finally, we tested the impact of low quality base filtering invoking a version of *V-Phaser* without NQS pre-processing filters. The positive control data were 454 read sets derived from an artificial mixture of eight strains of West Nile Virus (WNV) for which we knew the individual strain sequence. We limited our analysis to regions of the genome covered by all eight individual sequences. Differences among the individual consensus assemblies defined the WNV variant set; a total of 110 variants were defined. We scored any error call that *V-Phaser* made on this set of variants as a false negative, and any variant call as a true positive.

**Figure 4:**
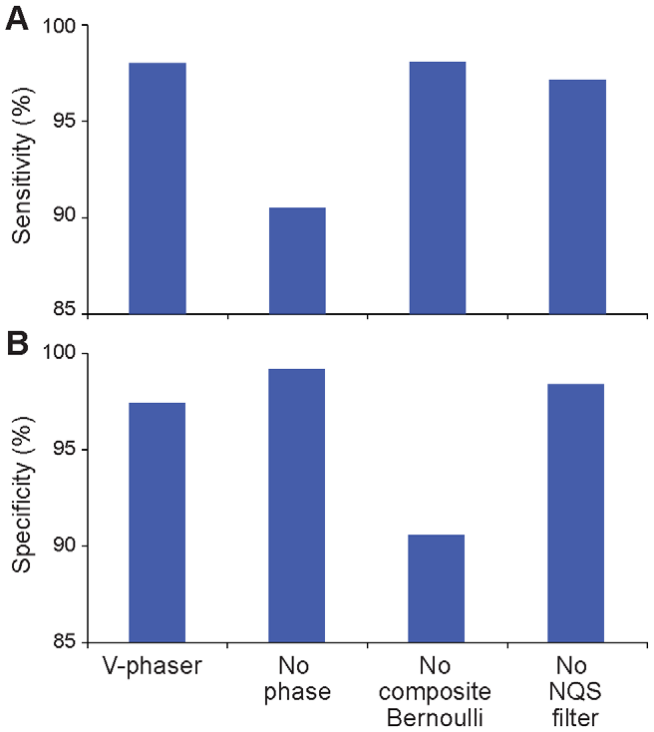
Phase information increased sensitivity, and base quality scores increased specificity. We compared *V-Phaser* to alternate versions of *V-Phaser* with specific components disabled. In the No Phase version, *V-Phaser* called variants without phase information. In the Uniform Errors version, *V-Phaser* estimated uniform error rates within homopolymer and nonhomopolymer regions without regard to assigned base qualities. In the No Filtering version, *V-Phaser* did not filter out low quality bases. (**A**) Phase information increased sensitivity. The version without phase information attained a sensitivity of 90%, but all other versions of *V-Phaser* used phase information and attained a sensitivity of 97% or more. We calculated sensitivity as the percentage of known variants correctly identified. Data are from WNV mixed population control dataset. (**B**) Individual base quality scores increased specificity. Among loci with mismatches, the Uniform Errors version had only 91% specificity, but all other versions incorporated base quality scores in their probability model and attained 97% specificity or more. We calculated specificity as the percentage of loci in the control sample correctly identified as having no variants among loci that had at least one candidate variant. Data are from infectious clone (HIV NL4-3) control dataset.

**Figure 5:**
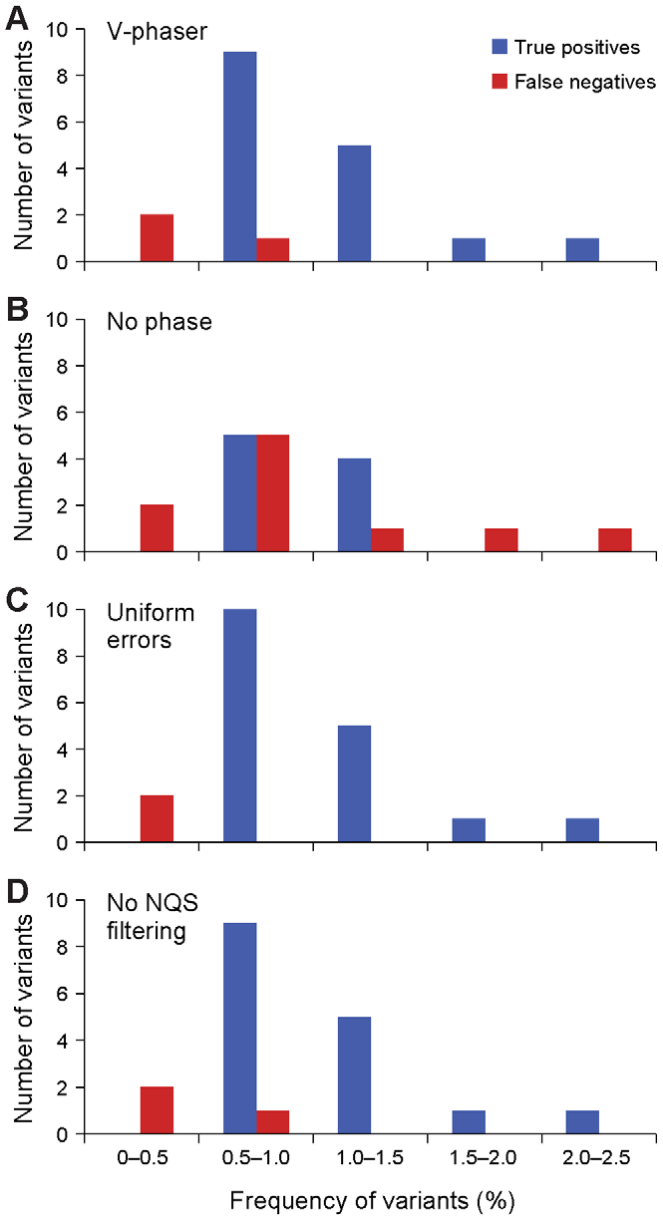
Phase information increased sensitivity to detect minor variants. Phase information increased sensitivity to detect low frequency variants, as shown by these histograms of variants under 2.5%. All versions of *V-Phaser* detected 100% of the variants above 2.5% frequency, so these variants are not shown here. All versions of *V-Phaser* with phase information (**A**), (**C**), and (**D**) detected most variants below 1% in frequency, but the No Phase version (**B**) missed many variants below 1% and some variants as high as 2.5%. Data are from control WNV mixed population.

Of the 110 variants in the WNV variant set, 102 variants were detected with frequencies ranging from 0.3% to 47.5%, and a median frequency of 11.3% (Table S1). Eight variants were not observed on any sequence reads. *V-Phaser* called 100/102 variants present in the data resulting in a sensitivity of 98% (Figure 4a), including 15/17 (88%) of the variants at frequencies under 1% (Figure 5 a–d). All versions of *V-Phaser* could detect 100% of the variants above 2.5%, but without *phased VDTFs V-Phaser* could detect only 9/17 (53%) of the minor variants present at less than 1.0% in frequency, and it still missed other variants with frequencies as high as 2.4%. Of the remaining 10,004 loci assumed to be non-variant based on consensus sequence comparison, *V-Phaser* called 143 variants, for a putative specificity of 99%. Out of 555 loci that showed variation in the mixture read set but not in the parental strains, *V-Phaser* correctly called 74% of them as errors. It is possible that many or most of the mistaken variant calls could be artificial variants or mutations that were introduced somewhere in the process of creating the mixture, rather than sequencing errors.

Because of the unknown rate of novel variants introduced during passage of the WNV samples, we used an infectious clone (HIV NL4-3) as a negative control to more accurately measure the specificity of *V-Phaser*. We scored any variant calls that *V-Phaser* made on the negative control as a false positive, and any error calls as a true negative. Among all loci in the negative control read set, 87% had no mismatches. Considering only the loci that harbored variation, all versions of *V-Phaser* maintained specificity greater than 97% if they incorporated individual base quality scores, but for the version using uniform errors, the specificity fell to 91% (Figure 4b). Among these sites with variation, *V-Phaser* called 29 sites that ranged in frequency from 0.4% to 5.6% as true variants; some of these sites may actually be biological variants and not process errors (see discussion below). If the composite Bernoulli model correctly described the error distribution, then 95% of the time *V-Phaser* would not make any false positive calls on the entire sample. Clearly, the composite Bernoulli model fits the error distribution better than a uniform error model, but the false positives are evidence that at least some errors did not follow the model.

**Figure 6:**
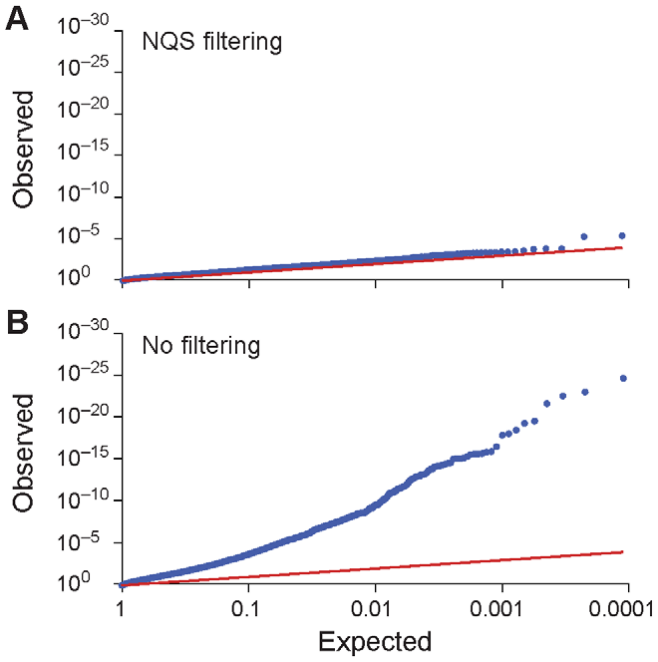
NQS filtering improves fit of probability model to data. (**A**) Quantile-quantile (q-q) plots under NQS filtering show good fit of the probability model to the observed distribution of errors. Since the probability model is discrete, p values are projected onto a uniform distribution, and the distribution of projected p values is compared with the expected null distribution. See Materials and Methods section for details. (**B**) In contrast, q-q plots under no filtering show that no filtering skews the calibration of the probability model used by *V-Phaser*. Q-q plots of models based on subsets of the reads demonstrate that this effect becomes more pronounced with increasing coverage (see Figure S1). Q-q plots are scaled to fit curve, so y = x line is not at a 45 degree angle.

We tested the validity of the composite Bernoulli model by assessing how well the model fit the error distribution with and without filtering using a quantile-quantile (q-q) plot as described in the Materials and Methods section. Compared to the unfiltered data, the filtered data produced a model that fit the observed error distribution better (Figure 6 a–b). Without pre-processing filters, *V-Phaser* systematically overestimated the probability of error. This overestimation of the model seemed to be a function of the number of low quality bases. As we sampled without replacement from 1% to 100% of the reads, we saw an increasing skew in the q-q plot (Figure S1 a–f). In addition, to test whether homopolymer related artifacts in 454 sequencing were causing *V-Phaser* to overcall variants, we examined the error calls made by *V-Phaser* on the clonal HIV NL4-3 data. Since homopolymer related artifacts systematically violate model assumptions, the resulting overcalls would be expected to cluster in or near homopolymer regions. False positives were not significantly more likely to be observed in homopolymer nucleotide runs, nor in regions proximal to these runs, as compared to residues outside of these regions, regardless of whether a variant is called with phase or without phase (χ^2^ test p>0.4 in all comparisons). These results were consistent if we extended the homopolymer flanking regions to three or four instead of two bases. Therefore, false positives appear to be unrelated to homopolymer related artifacts and *V-Phaser* appears to have no strong susceptibility to errors induced by system error on the 454 sequencing platform.

### Comparison to other variant calling algorithms

We ran several other variant calling programs on our control data sets. The programs *ShoRAH* [12] and *ViSPa* [25] both generated compute errors that were not easily resolved by the software's authors when run on our data set. We successfully ran *Segminator II* [21] and *QuRe* [13]. *QuRe* filters regions of the genome that have less than 30-fold read coverage or are below the 5^th^ percentile of coverage (defaults); as such comparison of sensitivity and specificity across the various algorithms was computed only across the bases interrogated by *QuRe*. Sensitivity and specificity of the three programs, in addition to results for *V-Phaser* with phasing turned off, are shown in Table 1. *V-Phaser* outperformed both of the other programs in specificity. Although *Segminator II* had 100% sensitivity, it achieved this at the expense of a very high false positive rate, calling variants at more than 20% of the examined invariant sites. Notably, the counting of inserted or deleted bases as false positives can significantly impact reported specificities. *V-Phaser* reports a deleted base in two instances while *QuRe* and *Segminator II* report 841 and six respectively. Inclusion of indels in the measure of specificity decreases *QuRe*'s specificity considerably (Table 1), but this likely a less accurate measure of the algorithms specificity since such errors could be easily filtered.

**Table 1:** Comparison of *V-Phaser* to other viral variant callers.

Program	*Segminator II*	*QuRe*	*V-Phaser*	*V-Phaser* (no phasing)
Sensitivity	100.0%	89.0%	96.0%	89.0%
Specificity	88.% (88.8%)	97.1% (88.7%)	99.9% (99.9%)	99.7% (99.6%)

Sensitivities and specificities reported across residues interrogated by all programs. Sensitivity is measured as the fraction of the known variants found by each program in the WNV mixed population control data set. Specificity is the fraction of sites not containing known variants that were called as invariant in the HIV NL4-3 control data set; values reported in parentheses include inserted and deleted bases (see Materials and Methods).

### Applying *V-Phaser* to clinical data

**Figure 7:**
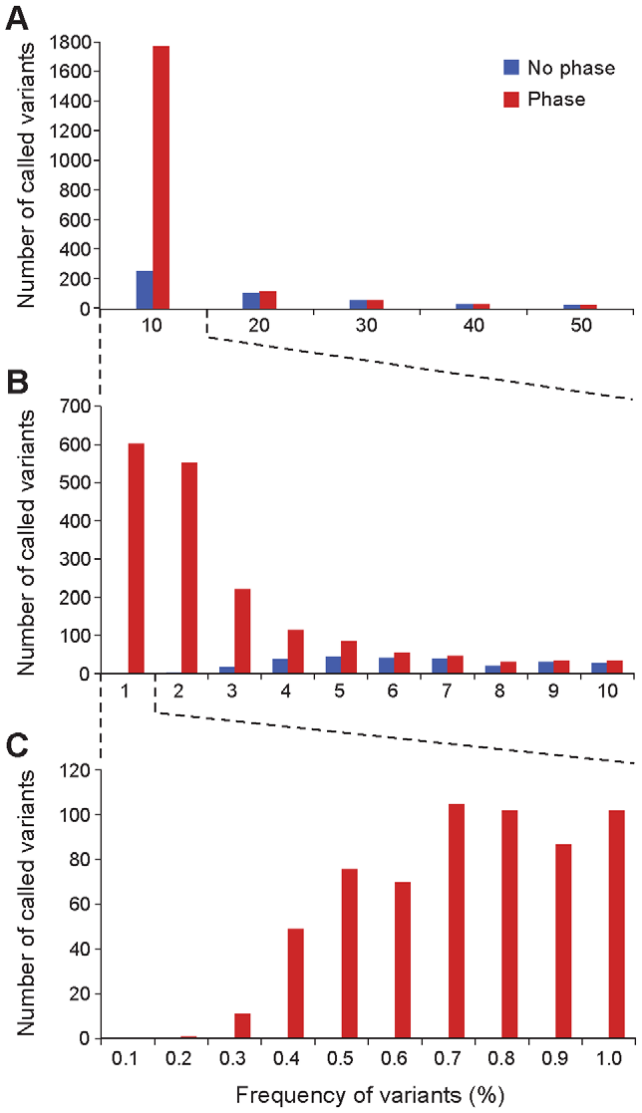
Low frequency variants overwhelmingly called with phase. Histogram shows low frequency variants overwhelmingly called with phase thresholds. Variants frequencies are estimated by the frequencies of variants among the reads at that position. Versions of *V-Phaser* with and without phase thresholds called variants on a clinical sample that are binned by their frequency at their locus. Most variants <5% were detected only be *V-Phaser* with phase thresholds, and the version without phase thresholds detected no variants <1%.

We applied *V-Phaser* to data from an individual with chronic HIV-1 infection taken from a larger study [24], and we analyzed called variants by the frequency of these variants among the reads at that position. Using just *unphased VDTFs*, *V-Phaser* called only 485 variants, none of which were <1%; using no filtering, *V-Phaser* called 1,778 variants; with *phased VDTFs* and filtering, *V-Phaser* detected 2,015 variants, including 603 variants with frequency <1% (Figure 7). Notably, *V-Phaser* detected variants down to 0.2%, a detection threshold comparable to allele-specific PCR [26]. More than one out of every five loci had a recognized variant. Assuming the specificity of *V-Phaser* remained constant, the positive predictive value was estimated as 98%. Of note, *V-Phaser* identified 42 insertions or deletions (indels) as variants, and *V-Phaser* detected 198 triallelic loci and 19 quadallelic loci.

## Discussion

*V-Phaser* called rare variants in the presence of error in massively parallel sequencing data of highly diverse viral populations with >97% sensitivity and >97% specificity. Applied to a sample from a chronically HIV-1 infected individual, *V-Phaser* could identify as many as 603 minor variants at population frequencies of <1%. These variants were detected without *a priori* knowledge of the specific mutations, and biological variants at frequencies as low as 0.2% were identified, comparable to the detection threshold of allele-specific PCR, which is restricted to assaying known mutations. In these data, *V-Phaser* called 42 indels and identified more than 200 loci (roughly 2% of the genome) with more than one variant.

In direct comparisons to two other recently published variant callers, *Segminator II* [21] and *QuRe* [13], *V-Phaser* outperformed both algorithms on specificity and outperformed *QuRe* on both specificity and sensitivity. This is not surprising since *QuRe* implements the error correction model of Wang *et al.* [27], which only considers pileup information. In fact, if we turn off the phasing portion of *V-Phaser*, it performs identically to *QuRe* on sensitivity, but even better on specificity (Table 1). *Segminator II* identifies all the true variants in the WNV mixed population data set, but at the cost of an unacceptably high false positive rate. Its alignment-based read filtering only removes errors arising from process-based indels while ignoring errors from other sources, such as random substitution errors due to sequence misreads or PCR errors in library construction.

The comprehensive *V-Phaser* model clearly outperformed the model using uniform error rates, but the number of false positives was higher than expected with the Bonferroni correction. Some of these false positives detected in the negative-control might actually be low level variants present in the HIV NL4-3 cDNA libraries used to generate the read set that had not been previously detected. Some might be errors introduced early and amplified to create correlated errors not modeled by *V-Phaser*. Particularly when considering the 454 sequencing process, which generates systematic errors in regions around homopolymers, correlated error may occur in such regions. However, we have multiple reasons to believe that this has a small impact on the final *V-Phaser* calls. First, *V-Phaser*'s base context model in the quality recalibration accounts for some amount of homopolymer error. Second, our *ReadClean454* read cleanup step during the alignment phase removes or marks as low quality the majority of errors derived from homopolymer misreads or “carry forward and incomplete extension” (CAFIE) errors (a related 454 error mode) [24]. Third, we examined the error calls made by *V-Phaser* on the clonal HIV NL4-3 data to see if they clustered in or near homopolymer regions, and we found them to be randomly distributed with respect to homopolymer regions. While the false positive rate was very low for the highly diverse clinical sample we used, it could be higher for samples with very low diversity since the false positive rate is inversely proportional to the total number of true variants. Another potential weakness of the model is the modeling of indels. DePristo et al. [17] suggest that indel errors distribute differently from other errors and need to be modeled differently. We did not explicitly test *V-Phaser* for indel detection in the artificial mixture used as the positive control, since the set of variants had no indels.

*V-Phaser* uses phase information to increase sensitivity. Correlated errors under the model are rarer than errors in general, making it easier to call correlated variants. One potential problem is the presence of chimeras, where one read is a composite from two different genomes. Chimeras can decrease the correlation between variants, but data from Hedskog, et al. [28] suggest that chimeras occur rarely, making it difficult to significantly obscure any correlation. The biggest limitation to using phase information is the read length generated by the sequencing platform. We saw that correlated variants need to be close enough to add to the sensitivity, and that this phase distance is bounded by the average read length (Figure 2). For the 454 platform the average read length is over 500 bp, but for other platforms the average read length is much shorter. This limitation could be overcome by utilizing paired-end reads to extend the phase distance to cover variants significantly farther apart.

*V-Phaser* is a variant calling tool that uses phase information to increase sensitivity and models base quality to increase specificity. *V-Phaser* is an effective tool to call variants in the presence of errors from massively parallel sequencing data with high specificity and high sensitivity. We designed *V-Phaser* to overcome specific challenges to calling variants in small, diverse viral genomes, but *V-Phaser* is general enough to analyze read sets from other populations as well, such as metagenomic data and tumor sequencing data, making it a novel algorithm with wide utility.

## Materials and Methods

### Ethics statement

The subject gave written informed consent and the study was approved by the Massachusetts General Hospital and granted exemption by the Massachusetts Institute of Technology Review Boards.

### Statistical model

We construct a composite model of independent Bernoulli random variables that are not identically distributed to allow error rates to vary from base to base. We suppose that the base *b_ik_* at genomic locus *i* and read *k* is measured at an error rate *p_ik_*, where reads are aligned to a reference assembly with loci numbered from 1 to *l*, and reads at locus *i* are numbered from 1 to *n_i_*, the coverage at locus *i*. We define the error random variable *E_ik_* to be 1 if *b_ik_* is measured incorrectly, and 0 otherwise. Let the random variable *X_i_* be the number of errors that occur at locus *i*:
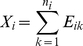
(1)


Under the special case that the errors are independent and identically distributed Bernoulli random variables, such that *p_ik_* = *p_i_* for all reads *k* at locus *i*, *X_i_* follows a binomial distribution, so the probability *f_i_*(*x*) that *x* or more errors occur at locus *i* with coverage of *n_i_* reads is as follows:
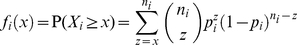
(2)


More generally, if *X_i_* is the sum of independent Bernoulli random variables that are not identically distributed, we can calculate *f_i_*(*x*) with the recursive application of the discrete convolution formula, where we define the random variable *U_ir_* as the number of errors that occur at locus *i* in the first *r* reads:
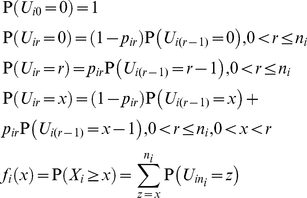
(3)


We define the unphased variant detection threshold *t_i_* as the smallest *t* such that *f_i_*(*t*) is statistically significant. To adjust for multiple testing, we use the Bonferroni correction since errors are uncorrelated under the null hypothesis. At a significance level α, and applying the Bonferroni correction for testing the total number of positions sequenced *c*, we calculate *t_i_* as follows:
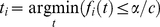
(4)


If mismatches at position *i* occurred in *t* reads, we can infer if the mismatches are variants by comparing *t* to *t_i_*. If *t* is greater than or equal to *t_i_*, then we infer that not all of the mismatches are errors, and at least one of them is a variant. If *t* is less than *t_i_*, then we infer that we cannot distinguish these mismatches from error.

Under the probability model, errors are independent, but variants can be phylogenetically related. Thus, we can also distinguish variants from errors if mismatches at one locus are correlated with mismatches at a different locus. In particular, we define the error random variable *E_ijk_* to be 1 if errors occur at both loci *i* and *j* on read *k* and 0 otherwise. Then the number of errors *X_ij_* that occur in phase at both loci *i* and *j*, with shared coverage of *n_ij_* reads that span both *i* and *j*, is the sum of error random variables as before. In the special case that errors are identically distributed Bernoulli random variables, where *p_ik_* = *p_i_* and *p_jk_* = *p_j_* for all reads *k* that cover both loci *i* and *j*, we calculate the phase probability *g_ij_*(*x*) as follows:
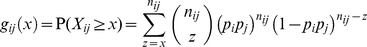
(5)


In the more general case, we can calculate *g_ij_*(*x*) by recursively applying the discrete convolution formula as before, where we define the random variable *U_ijr_* as the number of reads with errors that occur at locus *i* and locus *j* among the first *r* shared reads:
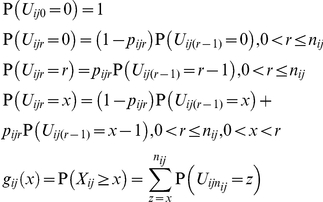



At a significance level α and applying the Bonferroni correction for testing the total number *b* of pairs of loci *i* and *j* such that *g_ij_* is defined, we can calculate the phased variant detection threshold *t_ij_* as follows:
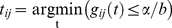
(6)


If we find phased mismatches at locus *i* and locus *j* on *t* reads, we can infer if they are variants by comparing *t* to *t_ij_*. If *t* is greater than or equal to *t_ij_*, then we infer that not all of these mismatches are errors. Otherwise, we cannot distinguish these mismatches from errors.

We define the *unphased variant detection threshold frequency* (*VDTF*) *F_i_* to be the frequency at which we begin to distinguish variants from errors at locus *i* and depth *n_i_*. Similarly, we define the *phased VDTF F_ij_* to be the frequency at which we begin to distinguish phased variants from errors at loci *i* and *j* and shared depth *n_ij_*. We calculate *F_i_* and *F_ij_* as follows:
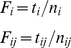
(7)


### Sample preparation, assembly, and annotation of control and sample read sets

We sequenced an HIV infectious clone (NL4-3) to serve as a negative control for our validations and HIV RNA derived from a clinical sample as previously described [24]. We derived the positive control read set from eight individual primary WNV strains isolated from mosquitoes and birds. Individual strains were passaged once in C6/36 cells for amplification, and equal concentrations of each strain were then pooled and used to infect C6/36 cells at a multiplicity of infection of 0.1. Viral RNA was isolated from these cultures (QIAmp viral RNA mini kit, Qiagen) and the RNA genome reverse transcribed to cDNA using Superscript III reverse transcriptase (Invitrogen), random hexamers (Roche) and a specific oligonucleotide targeting the 3′ end of the target genome sequences. Four overlapping PCR products, each of size ∼3 kb, were designed to capture the WNV coding region. PCR products were then pooled and sheared prior to library construction.

To generate each read set, whole viral genomes were sequenced using the Broad Institute's viral genome sequencing and assembly pipeline. Pooled PCR products (∼3 kb) were amplified using primer sets specific to either HIV or WNV, acoustically sheared, and sequenced on the 454 Genome Sequencer FLX Titanium (Roche) using standard protocols. The library was loaded into a picotiter plate (PTP) to yield >200-fold read coverage. Resulting sequence reads were trimmed of primer sequences, filtered for high quality, assembled *de novo* and annotated using the Broad Institute's *AssembleViral454* algorithm [24] and an in-house annotation algorithm. Reference consensus assemblies used in analyses are available from NCBI's GenBank under accessions HQ505665, JN819311, JN819312, JN819313, JN819315, JN819318, JN819319, JN819320, JN819315, JQ403053, and JQ403055; read data are available from NCBI's Short Read Archive (Project Accessions SRA045000 and SRA045569).

### Alignment and preprocessing filters

Once we generated the sequence data, we aligned and processed them using *ReadClean454* algorithm as previously described [24]. In particular, the algorithm corrects typical errors introduced by the 454 sequences, including carry forward and incomplete extension errors, homopolymer errors, and indels that break the open reading frame (ORF). Any base rearrangements do not affect the assigned base quality. Any insertions to preserve the ORF consist of N bases with an assigned base quality of 1.

We then flagged each base to indicate if it passed the NQS criteria [29]. A base met NQS criteria if its quality score was 20 or higher and the five bases to either side all had quality scores of 15 or higher. We omitted the final NQS criterion that at least nine of the ten flanking bases were perfect matches, since we expected the HIV genome to be variable enough that variants among the flanking bases could be relatively common [24]. For calling variants, *V-Phaser* ignored any bases flagged as not meeting the NQS criteria.

### Estimation of model parameters by EM algorithm

Once we aligned and preprocessed the sequence data, we estimated the model parameters and applied the model to the data to call variants. In the uniform error case, we estimated error rates in homopolymer and nonhomopolymer regions, where homopolymer regions are defined by runs of 3 or more identical nucleotides in a row. In the general case, we estimated error rates overall, per base transition (where each transition was a dinucleotide consisting of a base and its preceding base in the read sequence), and per read position (distance from the start or end of the read, whichever was closer). We then used these estimates in calibration equations [17] to estimate the error rate for each base.

As *V-Phaser* called variants, it iteratively adjusted model parameters using an EM algorithm. It initialized the algorithm by treating all mismatches as errors to estimate error rates. In the E step, it derived phased and unphased thresholds, called variants, and removed these variants from the list of errors. In the M step, it updated error rates due to the removal of called variants from the error list. *V-Phaser* continued to iterate until it could call no more variants.

### Correlation of *V-Phaser* errors with homopolymers

To test whether homopolymer-related artifacts in 454 sequencing which violated the model assumptions were causing *V-Phaser* to overcall variants, we divided the reference sequence of the clonal HIV NL4-3 genome into three categories: homopolymeric regions (defined as 3 or more of the same nucleotide in a row), homopolymer flanking regions (defined as 2 bases to either side of an homopolymeric region, representing the region in which CAFIE errors are expected to occur), and non-homopolymeric regions (the remainder of the genome). We then assigned each of the false positive calls made by *V-Phaser* to one of these categories and used a χ^2^ test (2 d.f.) to determine whether any region had more variants than expected.

### Construction of quantile-quantile plots

We use quantile-quantile (q-q) plots to assess the fit of our probability models under the null. To assess the fit of the model to the observed data, we compute the probability of observing each datum under the model using *F*(*x*), the cumulative distribution function (CDF), and compare the distribution of these observed probabilities against the expected distribution of cumulative probabilities under the null. For random variables with continuous CDFs, the expected distribution under the null is the uniform distribution between 0 and 1. For our probability models, the expected distribution of probabilities under the null is more difficult to calculate, since the CDF is discrete and varies from locus to locus with the base qualities at that locus. Since our models use discrete rather than continuous random variables, we redistribute the mass of the probability mass function to construct a uniform distribution. We define *G*(*x*), a projection of the cumulative distribution function (PCDF) that maps the CDF probabilities onto the uniform distribution in the following way:
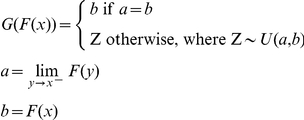
Conceptually, the PCDF redistributes massed probabilities uniformly to bridge discontinuities in the CDF. For example, if *X* is a random Bernoulli variable with success probability *p* and failure probability *q* = 1−*p* and CDF *F_X_*, then *F_X_*(*k*) = 0 for *k*<0, *F_X_*(*k*) = *q* for 0≤*k*<1, and *F_X_*(*k*) = 1 for *k*≥1. The discontinuities at *k* = 0 and *k* = 1 correspond to the massed probabilities for *X* at those values. *G* redistributes this mass uniformly to bridge the discontinuity. So whenever *X* = 0, *G*(*F_X_*(*k* = 0)) uniformly takes on a value between 0 and *q*, and whenever *X* = 1, *G*(*F_X_*(*k* = 1)) uniformly takes on a value between *q* and 1. *X* can take on no other values, so *G*(*F_X_(k* = *X*)) follows a uniform distribution between 0 and 1. So if we have *n* observations of *X*, about *p*/*n* of them will be 1 and about *q*/*n* of them will be 0. The expected cumulative probabilities for each observed 0 will all be *q*, but their projected probabilities will be uniformly distributed between 0 and *q*. Similarly, the projected probabilities for each observed 1 will be uniformly distributed between *q* and 1. If we sort the observations by their projected probabilities, then the projected probability for the *i*th observation will be very close to *i*/*n*. By construction, these projected probabilities are uniformly distributed between 0 and 1 under the null. So even though the CDFs vary by locus with the mix of error rates among bases at that locus, the PCDF remains uniform. Thus, we can compare the projected distribution of PCDF probabilities against the expected distribution under the null, which by design is the uniform distribution.

### Comparison to other variant callers

We evaluated *V-Phaser*'s performance in terms of sensitivity and specificity to detect variants relative to four other programs designed for variant detection in viral quasispecies populations: *ShoRAH* [12], *ViSPa* [25], *Segminator II* [21], and *QuRe* [13]. All programs were run according to standard parameters defined by the software authors. In all cases, we used the alignment and read cleaning (if any) methods recommended by the authors. Only the *Segminator II* and *QuRe* software packages successfully ran on our datasets. For *V-Phaser*, we used our standard process including *ReadClean454* [24] to error correct and align the reads. In all cases, we used our sample-specific consensus assemblies as the reference for alignment. Sensitivity was computed using our WNV mixed population control data set and specificity was determined using the HIV NL4-3 infectious clone control data set. In scoring the resulting calls, we ignored all inserted and deleted bases called (6 by *Segminator II*, 2 by *V-Phaser*, and 841 by *QuRe*), because we could not determine the exact number of discrete indel events called by *QuRe* and felt that it would be unfair to count all 841 as errors since such errors could be filtered (the data have no known indels based on the input strain sequences).

## Supporting Information

Figure S1 **Impact of not filtering by NQS on model calibration with increased coverage.** Quantile-quantile (q-q) plots for no NQS filtering data model show that the skew in the calibration of the probability model used by *V-Phaser* increases with increased sequence coverage. The impact of the skew is demonstrated for (**A**) 5-fold, (**B**) 27-fold, (**C**) 52-fold, (**D**) 131-fold, (**E**) 262-fold, and (**F**) 528-fold sequence coverage.
(TIF)

Table S1 ***V-Phaser*** **variant calls in experimental WNV mixed population.** Eight parental strains of WNV were mixed at equal proportions and then infected into mosquito cells and allowed to proliferate, resulting in a final mixture with ratios set by the relative replicative success of the strains. The nucleotide sequence in each parental strain at residues known to contain a mutation are shown and a “.” indicates the strain has the dominant allele at that particular residue. Dominant residues are noted in the variant column. The true proportion of the parental strains in the sequenced mixture is not set, but since we know the strain or strains of origin for all of the variants, we can infer the mix of parental strain proportions that maximizes the likelihood of observing the actual counts (including zero) of all parental alleles in the sequencing data. The resultant frequencies are presented in the “expected” column to provide an estimate of the true frequency of the variants in the population. This allows us to capture the full effect of stochastic variation in the sequencing process on our ability to detect variants of any given population frequency.
(PDF)
