# Supplementary figures and images for: Highly Sensitive and Specific Detection of Rare Variants in Mixed Viral Populations from Massively Parallel Sequence Data
Source: PLoS Comput Biol. 2012 Mar 15;8(3):e1002417. doi: 10.1371/journal.pcbi.1002417 (PMC3305335; doi:10.1371/journal.pcbi.1002417)

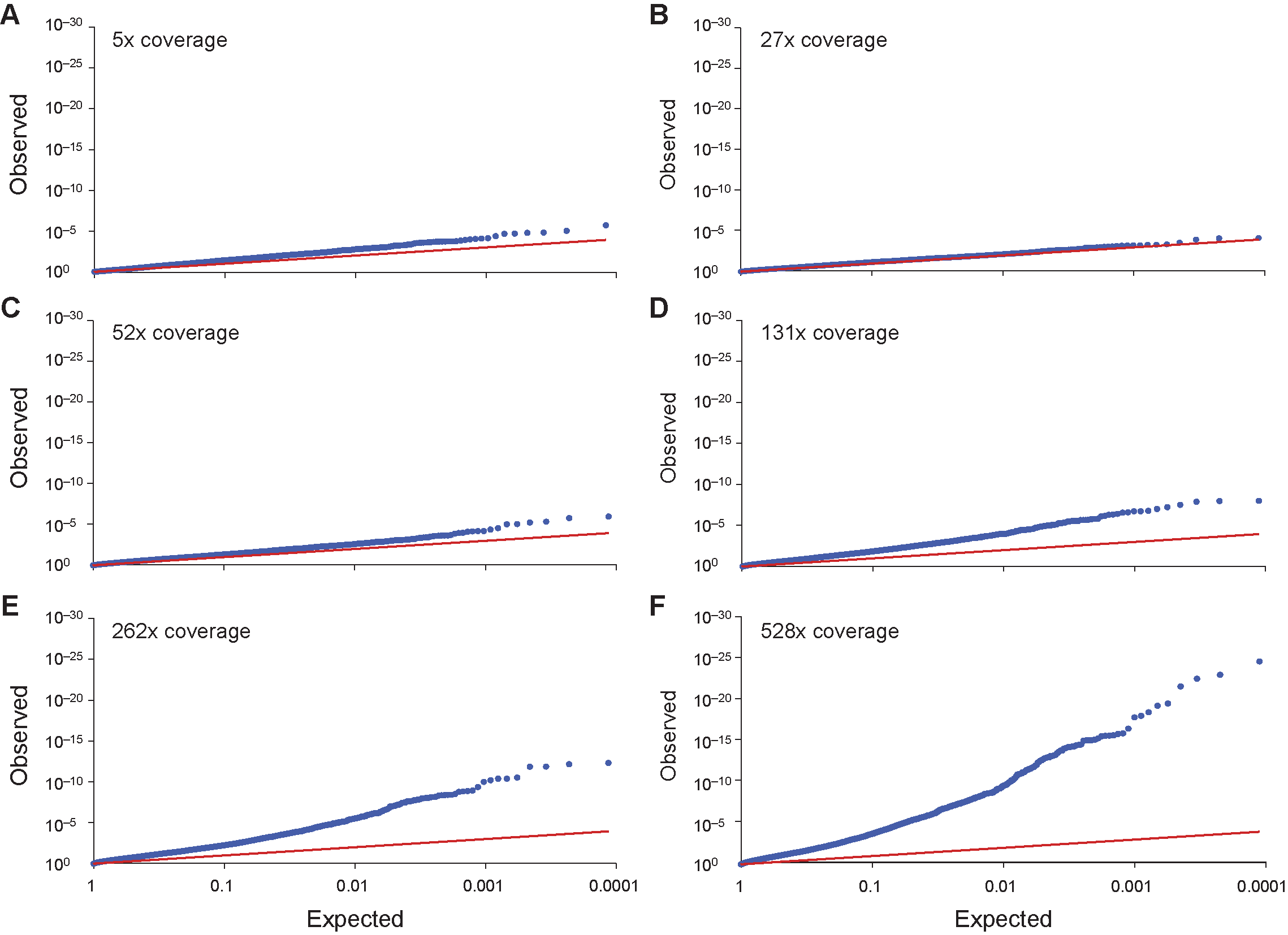

Supplement: Figure S1 — Impact of not filtering by NQS on model calibration with increased coverage. Quantile-quantile (q-q) plots for no NQS filtering data model show that the skew in the calibration of the probability model used by V-Phaser increases with increased sequence coverage. The impact of the skew is demonstrated for (A) 5-fold, (B) 27-fold, (C) 52-fold, (D) 131-fold, (E) 262-fold, and (F) 528-fold sequence coverage. (TIF) [file pcbi.1002417.s001.tif]
