# Supplementary material for: Highly Sensitive and Specific Detection of Rare Variants in Mixed Viral Populations from Massively Parallel Sequence Data
Source: PLoS Comput Biol. 2012 Mar 15;8(3):e1002417. doi: 10.1371/journal.pcbi.1002417 (PMC3305335; doi:10.1371/journal.pcbi.1002417)
Supplement: Table S1 — V-Phaser variant calls in experimental WNV mixed population. Eight parental strains of WNV were mixed at equal proportions and then infected into mosquito cells and allowed to proliferate, resulting in a final mixture with ratios set by the relative replicative success of the strains. The nucleotide sequence in each parental strain at residues known to contain a mutation are shown and a “.” indicates the strain has the dominant allele at that particular residue. Dominant residues are noted in the variant column. The true proportion of the parental strains in the sequenced mixture is not set, but since we know the strain or strains of origin for all of the variants, we can infer the mix of parental strain proportions that maximizes the likelihood of observing the actual counts (including zero) of all parental alleles in the sequencing data. The resultant frequencies are presented in the “expected” column to provide an estimate of the true frequency of the variants in the population. This allows us to capture the full effect of stochastic variation in the sequencing process on our ability to detect variants of any given population frequency. (PDF) [file pcbi.1002417.s002.pdf]

Highly sensitive and specific detection of rare variants in mixed viral populations from massively parallel sequence data

Alexander R. Macalalad, Michael C. Zody, Patrick Charlebois, Niall J. Lennon, Ruchi M. Newman, Christine M. Malboeuf, Elizabeth M. Ryan, Christian L. Boutwell, Karen A. Power, Doug E. Brackney, Kendra N. Pesko, Joshua Z. Levin, Gregory D. Ebel, Todd M. Allen, Bruce W. Birren, Matthew R. Henn

Table S1. V-Phaser variant calls in experimental WNV mixed population.

Variant	Strain1	Strain2	Strain3	Strain4	Strain5	Strain6	Strain7	Strain8	Expected Frequency*	Observed Frequency	V-Phaser	No Phase	No Composite Bernoulli (i.e. Uniform)	No NQS Filter
4674C	.	.	.	.	.	.	.	T	0.007	0.000	None	None	None	None
6433C	.	.	.	.	.	.	.	T	0.007	0.000	None	None	None	None
8079C	.	.	.	.	.	.	.	T	0.007	0.000	None	None	None	None
8295A	G	.	.	.	.	.	.	.	0.137	0.000	None	None	None	Variant
8301C	.	.	.	.	.	.	.	T	0.007	0.000	None	None	None	None
8751C	.	.	.	.	.	.	.	T	0.007	0.000	None	None	None	None
9360T	.	.	.	.	.	.	.	A	0.007	0.000	None	None	None	None
9537C	.	.	.	.	.	.	.	T	0.007	0.000	None	None	None	None
3774C	.	.	.	.	.	.	.	T	0.007	0.003	Error	Error	Error	Error
3625A	.	.	.	.	.	.	.	T	0.007	0.004	Error	Error	Error	Error
7938C	.	.	.	.	.	.	.	T	0.007	0.006	Variant	Error	Variant	Variant
1442C	.	.	.	.	.	.	.	T	0.007	0.006	Variant	Variant	Variant	Variant
4146G	.	.	.	.	.	.	.	A	0.007	0.007	Variant	Variant	Variant	Variant
7785C	.	.	.	.	.	.	.	T	0.007	0.007	Variant	Error	Variant	Variant
6243A	.	.	.	.	.	.	.	G	0.007	0.008	Variant	Variant	Variant	Variant
2872G	.	.	.	.	.	.	.	A	0.007	0.008	Variant	Error	Variant	Variant
1492T	.	.	.	.	.	.	.	C	0.007	0.008	Variant	Variant	Variant	Variant
9352T	.	.	.	.	.	.	.	C	0.007	0.009	Variant	Error	Variant	Variant
5709C	.	.	.	.	.	.	.	T	0.007	0.009	Variant	Error	Variant	Error
7380G	.	.	.	.	.	.	.	A	0.007	0.010	Variant	Variant	Variant	Variant
6426T	.	.	.	.	.	.	.	C	0.007	0.011	Variant	Variant	Variant	Variant
2466T	.	.	.	.	.	.	.	C	0.007	0.013	Variant	Variant	Variant	Variant
4803T	.	.	.	.	.	.	.	C	0.007	0.013	Variant	Variant	Variant	Variant
6996T	.	.	.	.	.	.	.	C	0.007	0.013	Variant	Error	Variant	Variant
3270A	.	.	.	.	.	.	.	G	0.007	0.014	Variant	Variant	Variant	Variant
10341C	.	.	.	.	.	.	.	T	0.007	0.016	Variant	Error	Variant	Variant
7270T	.	.	.	.	.	.	.	C	0.007	0.024	Variant	Error	Variant	Variant
6741C	.	T	.	.	.	.	.	.	0.086	0.030	Variant	Variant	Variant	Variant
4164C	.	.	.	.	.	.	T	.	0.070	0.034	Variant	Variant	Variant	Variant
2674G	.	.	.	.	.	.	A	.	0.070	0.037	Variant	Variant	Variant	Variant
3300C	.	.	.	.	.	.	T	.	0.070	0.045	Variant	Variant	Variant	Variant
2904T	.	.	.	.	.	.	C	.	0.070	0.055	Variant	Variant	Variant	Variant
6780C	.	.	.	.	T	.	.	.	0.096	0.057	Variant	Variant	Variant	Variant
7518G	.	.	.	.	.	.	A	.	0.070	0.058	Variant	Variant	Variant	Variant
6060C	.	.	.	.	T	.	.	.	0.096	0.060	Variant	Variant	Variant	Variant
5544G	.	.	.	.	.	.	T	.	0.070	0.063	Variant	Variant	Variant	Variant
999C	T	.	.	.	.	.	.	.	0.137	0.064	Variant	Variant	Variant	Variant
666T	.	.	.	.	.	.	C	.	0.070	0.065	Variant	Variant	Variant	Variant
7320C	.	.	.	.	T	.	.	.	0.096	0.067	Variant	Variant	Variant	Variant
6964T	.	.	.	.	C	.	.	.	0.096	0.070	Variant	Variant	Variant	Variant
1599C	.	.	.	.	T	.	.	.	0.096	0.073	Variant	Variant	Variant	Variant
4882G	.	.	.	.	.	.	A	.	0.070	0.075	Variant	Variant	Variant	Variant
3556T	.	.	.	.	.	.	C	.	0.070	0.076	Variant	Variant	Variant	Variant
2922T	.	.	C	.	.	.	.	.	0.098	0.079	Variant	Variant	Variant	Variant
6871G	.	.	.	.	A	.	.	.	0.096	0.079	Variant	Variant	Variant	Variant
5940C	.	.	.	.	.	.	T	.	0.070	0.081	Variant	Variant	Variant	Variant
2934G	.	.	A	.	.	.	.	.	0.098	0.082	Variant	Variant	Variant	Variant
7548G	.	T	.	.	.	.	.	.	0.086	0.083	Variant	Variant	Variant	Variant
8766C	.	.	T	.	.	.	.	.	0.098	0.089	Variant	Variant	Variant	Variant
1779T	.	C	.	.	.	.	.	.	0.086	0.091	Variant	Variant	Variant	Variant
6228G	.	.	.	.	.	.	A	.	0.070	0.092	Variant	Variant	Variant	Variant
4017C	.	.	.	.	.	.	T	.	0.070	0.093	Variant	Variant	Variant	Variant
6162A	.	.	G	.	.	.	.	.	0.098	0.094	Variant	Variant	Variant	Variant
6183C	.	.	T	.	.	.	.	.	0.098	0.095	Variant	Variant	Variant	Variant
1728A	.	.	.	.	G	.	.	.	0.096	0.099	Variant	Variant	Variant	Variant
10408C	.	.	.	.	.	.	T	T	0.077	0.100	Variant	Variant	Variant	Variant
9151C	T	.	.	.	.	.	.	.	0.137	0.100	Variant	Variant	Variant	Variant
6993T	.	.	.	.	.	.	C	.	0.070	0.103	Variant	Variant	Variant	Variant
10347T	C	.	.	.	.	.	.	.	0.137	0.103	Variant	Variant	Variant	Variant
5814T	.	.	C	.	.	.	.	.	0.098	0.103	Variant	Variant	Variant	Variant
7917C	.	.	.	.	T	.	.	.	0.096	0.104	Variant	Variant	Variant	Variant
5628G	.	.	.	.	A	.	.	.	0.096	0.105	Variant	Variant	Variant	Variant
3234T	.	.	.	.	C	.	.	.	0.096	0.107	Variant	Variant	Variant	Variant
573C	.	.	.	.	T	.	.	.	0.096	0.112	Variant	Variant	Variant	Variant
9687C	.	.	.	.	T	.	.	.	0.096	0.115	Variant	Variant	Variant	Variant

562C	.	.	.	.	T	.	.	.	0.096	0.116	Variant	Variant	Variant	Variant
2253A	G	.	.	.	.	.	.	.	0.137	0.117	Variant	Variant	Variant	Variant
9960T	.	.	.	.	C	.	.	.	0.096	0.121	Variant	Variant	Variant	Variant
5135T	.	.	.	.	C	.	.	.	0.096	0.125	Variant	Variant	Variant	Variant
6234A	.	G	.	.	.	.	.	.	0.086	0.125	Variant	Variant	Variant	Variant
3810C	.	.	.	.	T	.	.	.	0.096	0.128	Variant	Variant	Variant	Variant
3346C	.	.	.	A	.	.	C	.	0.146	0.132	Variant	Variant	Variant	Variant
6210C	T	.	.	.	.	.	.	.	0.137	0.133	Variant	Variant	Variant	Variant
9603A	.	.	G	.	.	.	.	.	0.098	0.133	Variant	Variant	Variant	Variant
9123C	.	.	.	T	.	.	.	.	0.146	0.134	Variant	Variant	Variant	Variant
1293C	.	.	.	.	.	T	.	.	0.146	0.135	Variant	Variant	Variant	Variant
6820C	.	.	.	T	.	.	.	.	0.146	0.136	Variant	Variant	Variant	Variant
5805T	.	.	.	C	.	.	.	.	0.146	0.136	Variant	Variant	Variant	Variant
7182C	.	.	.	T	.	.	.	.	0.146	0.136	Variant	Variant	Variant	Variant
4536G	.	.	.	A	.	.	.	.	0.146	0.141	Variant	Variant	Variant	Variant
7161C	.	.	.	T	.	.	.	.	0.146	0.142	Variant	Variant	Variant	Variant
7158A	.	.	.	G	.	.	.	.	0.146	0.142	Variant	Variant	Variant	Variant
7110C	.	.	.	T	.	.	.	.	0.146	0.143	Variant	Variant	Variant	Variant
7134C	T	.	.	.	.	.	.	.	0.137	0.146	Variant	Variant	Variant	Variant
6288C	.	.	.	T	.	.	.	.	0.146	0.148	Variant	Variant	Variant	Variant
1533C	T	.	.	.	.	.	.	.	0.137	0.149	Variant	Variant	Variant	Variant
8808C	T	.	.	.	.	.	.	.	0.137	0.150	Variant	Variant	Variant	Variant
6721G	.	.	.	A	.	.	.	.	0.146	0.150	Variant	Variant	Variant	Variant
6007C	.	.	.	T	.	.	.	.	0.146	0.151	Variant	Variant	Variant	Variant
1117G	.	.	.	A	.	.	.	.	0.146	0.157	Variant	Variant	Variant	Variant
7977A	G	.	.	.	.	.	.	.	0.137	0.157	Variant	Variant	Variant	Variant
3138T	.	.	.	C	.	.	.	.	0.146	0.160	Variant	Variant	Variant	Variant
6246T	.	.	.	C	.	.	.	.	0.146	0.169	Variant	Variant	Variant	Variant
3111G	A	.	.	.	.	.	.	.	0.137	0.173	Variant	Variant	Variant	Variant
7893C	.	.	.	.	.	T	.	.	0.146	0.175	Variant	Variant	Variant	Variant
9439G	A	.	.	.	.	.	.	.	0.137	0.178	Variant	Variant	Variant	Variant
4129C	T	.	.	.	.	.	.	.	0.137	0.179	Variant	Variant	Variant	Variant
3120G	A	.	.	.	.	.	.	.	0.137	0.186	Variant	Variant	Variant	Variant
5211A	.	.	.	.	G	.	.	.	0.096	0.186	Variant	Variant	Variant	Variant
7959C	.	.	.	.	T	.	.	.	0.096	0.193	Variant	Variant	Variant	Variant
5134A	.	.	G	.	G	.	.	.	0.194	0.233	Variant	Variant	Variant	Variant
6238C	T	.	T	.	.	.	.	.	0.235	0.236	Variant	Variant	Variant	Variant
9912C	.	T	.	.	.	T	.	.	0.232	0.239	Variant	Variant	Variant	Variant
483C	.	T	.	.	.	T	.	.	0.232	0.250	Variant	Variant	Variant	Variant
8283G	.	A	.	.	.	A	.	.	0.232	0.282	Variant	Variant	Variant	Variant
7221C	.	T	.	.	.	T	.	.	0.232	0.284	Variant	Variant	Variant	Variant
4389G	.	A	.	.	.	A	.	.	0.232	0.315	Variant	Variant	Variant	Variant
10400T	.	.	C	.	.	.	.	.	0.098	0.410	Variant	Variant	Variant	Variant
7878C	.	T	.	.	.	T	.	.	0.232	0.427	Variant	Variant	Variant	Variant
660T	.	C	.	.	.	C	C	C	0.309	0.475	Variant	Variant	Variant	Variant

* The eight parental strains were mixed at equal proportions and then infected into mosquito cells and allowed to proliferate, resulting in a final mixture with ratios set by the relative replicative success of the strains. Thus, we have not set the true proportion of the parental strains in the sequenced mixture. However, since we know the strain or strains of origin for all of the variants, we can infer the mix of parental strain proportions that maximizes the likelihood of observing the actual counts (including zero) of all parental alleles in the sequencing data. The resultant frequencies are presented in the "expected" column to provide a most likely measure of the true frequency of the variants in the population. This allow us to capture the full effects of stochastic variation in the sequencing process on our ability to detect variants of given population frequency.
